# Regulators of Oncogenic Mutant TP53 Gain of Function

**DOI:** 10.3390/cancers11010004

**Published:** 2018-12-20

**Authors:** Satomi Yamamoto, Tomoo Iwakuma

**Affiliations:** 1 Department of Cancer Biology, The University of Kansas Cancer Center, University of Kansas Medical Center, Kansas City, KS 66010, USA; syamamoto@kumc.edu; 2 Department of Hematology and Oncology, Children’s Mercy Research Institute, Kansas City, MO 64108, USA

**Keywords:** TP53, mutant TP53, gain of function, post-translational modification, molecular chaperone, single nucleotide polymorphism, dimer-forming mutation

## Abstract

The tumor suppressor p53 (TP53) is the most frequently mutated human gene. Mutations in TP53 not only disrupt its tumor suppressor function, but also endow oncogenic gain-of-function (GOF) activities in a manner independent of wild-type TP53 (wtp53). Mutant TP53 (mutp53) GOF is mainly mediated by its binding with other tumor suppressive or oncogenic proteins. Increasing evidence indicates that stabilization of mutp53 is crucial for its GOF activity. However, little is known about factors that alter mutp53 stability and its oncogenic GOF activities. In this review article, we primarily summarize key regulators of mutp53 stability/activities, including genotoxic stress, post-translational modifications, ubiquitin ligases, and molecular chaperones, as well as a single nucleotide polymorphism (SNP) and dimer-forming mutations in mutp53.

## 1. Introduction

The primary role of the tumor suppressor p53 (TP53) is to control cell cycle progression, senescence, DNA repair, cell death, and cell metabolism, leading to inhibition of tumorigenesis [1,2]. Compared to all other human genes, *TP53* has the highest mutation rate, the majority of which are missense mutations with amino acid changes mainly in the DNA binding domain. There are hotspot mutations including R175, G245, R248, R249, R273, and R282, accounting for ~30% of p53 mutations [3]. In human cancers, the presence of mutant TP53 (mutp53) is associated with advanced stages of disease, metastasis, recurrence, and patient’s poor prognosis, even when compared with TP53 deletion [4,5,6]. Such oncogenic activities of mutp53 are referred to as gain of function (GOF) [7]. Mutp53 GOF was first notified in 1993, when Dittmer et al. [8] observed that overexpression of R175H or R273H mutants in *TP53-null* Saos2 cells enhanced soft agar colony formation and tumor development in mice. Since then, considerable evidence has solidified that mutp53 not only loses wild-type TP53 (wtp53) function, but also enhances tumor malignancy in vitro and in vivo independent of wtp53. 

**Figure 1:**
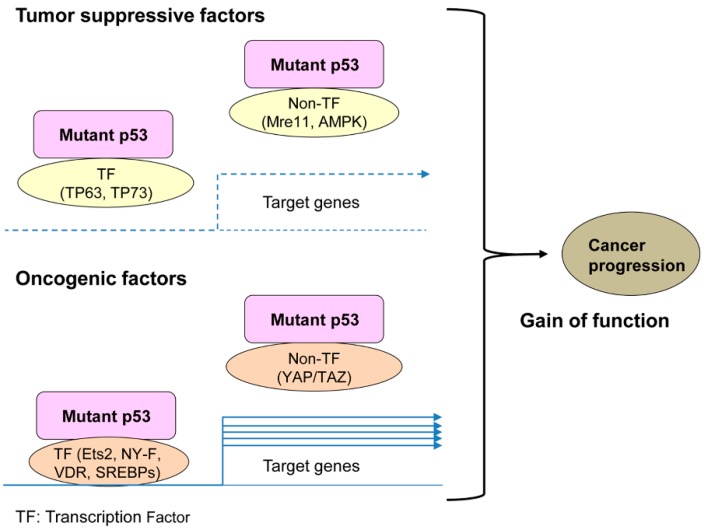
Mechanisms of mutant tumor suppressor p53 (mutp53) gain of function (GOF).

The oncogenic function of mutp53 is mainly caused by altered structure and properties of mutp53 to bind with other oncogenic (e.g., Ets2, SREBPs, vitamin D receptor, NF-Y, AMPK) or tumor suppressive (e.g., TP63, TP73, Mre11) proteins [7,9,10,11,12,13,14]. Many of the mutp53 binding partners are transcription factors (Figure 1). The mutp53-protein interaction allows mutp53 to alter the function of these binding partners, showing unexpected oncogenic activities in cells. Detailed description about mechanisms of GOF and mutp53 binding partners is well-documented in many other excellent review articles [8,11,15,16,17,18,19,20,21]. As an additional mechanism, through studies of mutp53-knockin mice, Lozano’s group demonstrate that mutp53 (mouse R172H, equivalent with human R175H) is inherently unstable, which can be accumulated by oncogenic and genotoxic stress similarly to wtp53, leading to enhanced cancer progression [22,23,24,25]. These studies strongly suggest that levels of mutp53 are crucial for the GOF activity. However, the mechanisms of GOF appear to be complicated and remain unsolved. This is mainly because not all TP53 mutants have the same activity and the biological properties of each mutp53 can be cellular context-dependent [15]. 

Generally, mutp53 can be roughly divided into two subtypes, DNA contact and conformational (or misfolded/unfolded) mutants. DNA contact mutants have mutations in amino acids where TP53 directly contacts with DNA, leading to impaired TP53’s binding activity while sparing the intact TP53 structure. On the other hand, conformational mutants robustly alter the TP53 structure and disrupt the DNA binding activity [26,27]. As mentioned above, accumulation of mutp53 plays a crucial role in the oncogenic GOF activity. The mutp53 protein is often stabilized in cancer cells, whereas wtp53 has a short half-life in both normal tissues and cancer cells [22,23,28]. Recently, our group has shown that cholesterol-lowering drugs, statins, induce some TP53 mutants for CHIP (carboxyl terminus of HSC70-interacting protein/Stub1) ubiquitin ligase-mediated degradation depending on their structures (misfolded or not), because conformational or misfolded mutants are more sensitive to statin treatment than wtp53 and DNA contact mutants [29]. Additionally, Maan and Pai [30] demonstrate that TP53 mutants prone to aggregation are ubiquitinated and degraded by CHIP in hypoxic conditions. Thus, mechanisms behind mutp53 stability and degradation can be dependent on the structure and/or biochemical properties.

Like wtp53, mutp53 stability is regulated by various genotoxic stresses, nutrient depletion, and oncogenic or tumor suppressive proteins [23,25], which is mediated by multiple E3 ubiquitin ligases (e.g., MDM2: Mouse double minute 2, CHIP, Pirh2) and molecular chaperones (e.g., heat shock proteins: HSPs including HSP90, HSP70, and HSP40). In addition, mutp53 GOF activities can be altered by post-translational modifications (PTMs), such as phosphorylation, acetylation, and ubiquitination, as well as by a single nucleotide polymorphism at codon 72 (SNP72) and dimer-forming mutations. Although many review articles describe mutp53-binding partners and their downstream signaling as GOF mechanisms [8,15,16,17,18,19,20,21], a few reviews address upstream factors and PTMs that alter mutp53 stability and its oncogenic GOF activities which are the main focus of this review paper. 

## 2. Mutp53 Stability and Activity Are Altered by Stress and Chemical Compounds

Mutations in TP53 impair the transcriptional activity (loss of function: LOF). The majority of TP53 mutations are missense mutations. These dysfunctional TP53 mutant proteins are present in the cells and retain or gain abilities to bind with other proteins, resulting in altered function of these binding partners to enhance tumor malignancy, referred to as GOF [7,9,10,11,12,13,14] (Figure 1). 

**Figure 2:**
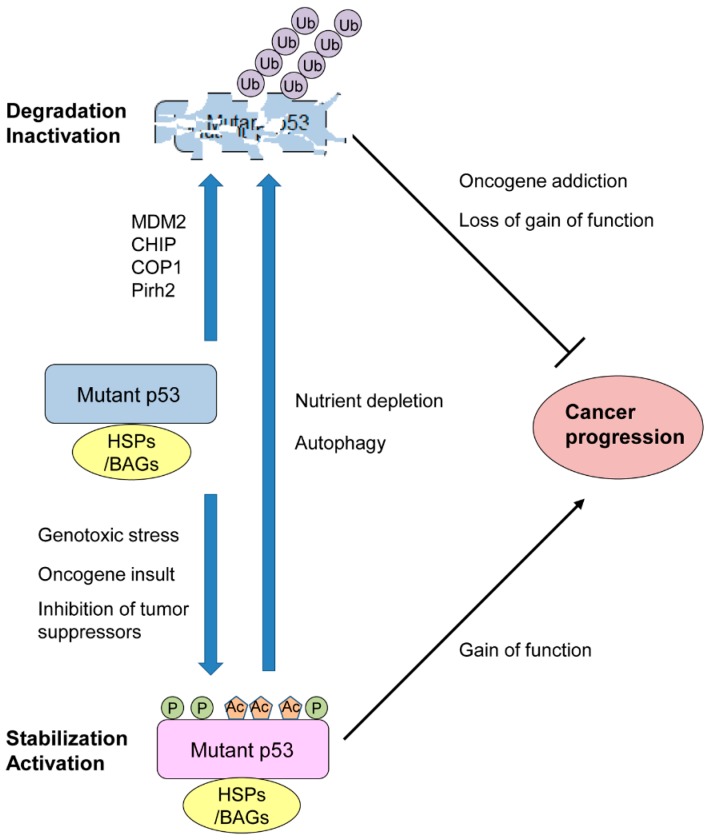
Environmental stresses alter stability and activity of mutant TP53 (mutp53) crucial for its oncogenic gain of function (GOF).

Due to mutp53’s inability to transactivate downstream targets including its ubiquitin ligase MDM2, it was considered that mutp53 could be accumulated in both cancer and normal tissues. However, studies using mutp53 knockin mice have demonstrated that mutp53 levels are low in most normal tissues, while mutp53 is accumulated in many cancer tissues [22,23,31]. These in vivo studies also reveal that accumulation of mutp53 contributes to mutp53 GOF, such as shorter survival, more metastasis, and increased frequency of carcinoma (epithelial origin) incidence as compared to those in *TP53^−/−^* mice [22,31]. Additionally, these studies and others show that inherently unstable mutp53 can be stabilized by genotoxic stress (e.g., ionizing radiation, reactive oxygen species), oncogenic insults (e.g., Myc, K-Ras, ErbB2), and loss or inhibition of other tumor suppressive proteins (e.g., p16INK4A, IL27RA, PML: Promyelocytic leukemia) (Figure 2) [23,25,32,33,34]. Since most of these studies are demonstrated using *TP53^R172H^*-knockin mice, it remains unclear whether levels of other TP53 mutants also increase by these environmental stresses in mice. 

Mutp53 can be degraded by cellular conditions associated with autophagy (Figure 2). Suppression of macroautophagy by spautin-1 under glucose free and confluent conditions induces degradation of mutp53 through chaperone-mediated autophagy (CMA) in a manner dependent on lysosome and independent of the ubiquitin–proteasome pathway [35,36]. Glucose restriction is also shown to induce deacetylation and degradation of multiple TP53 mutants (K132Q, R175H, L194F, R280K, R280T), which enhances activation of autophagic cell death and tumor suppression, since mutp53 inhibits autophagy (also mentioned in the acetylation section below) [36]. Furthermore, prolonged inhibition of the proteasome by MG132 treatment leads to degradation of multiple TP53 mutants (R175H, L194F, G245A, R280K), rather than stabilizing mutp53, via autophagy, since inhibition of autophagy by knocking down ATG5 and Ulk1 rescues MG132-induced mutp53 degradation [37,38]. Although these cellular stresses alter stability of mutp53, detailed molecular mechanisms remain to be clarified. Table 1 summarizes these regulators that directly alter mutp53 levels and/or its GOF activities.

By mainly focusing on the involvement of PTMs, specific chaperones, and ubiquitin ligases in mutp53 stability/activity, we briefly discuss chemical compounds that can alter mutp53 stability/activity. These include mutp53 accumulators (e.g., DNA damage agents), degraders/depleters (e.g., arsenic compounds, statins, HSP90 inhibitors, histone deacetylase inhibitor: HDACi, spautin-1, gambogic acid, phenformin), and reactivators (e.g., PRIMA-1, APR-246, MIRA1/2/3, STIMA-1, RITA, NSC319726/ZMC1, chetomin, stictic acid, p53R3, SCH529074, WR-1065, thiosemicarbazones, PhiKan083, CP-31398, PK7088) [7,39,40,41,42]. 

DNA damage agents result in increased levels of mutp53 to alter function of TP53-binding partners, which contributes to chemoresistance [24,43,44,45,46]. For example, a chemotherapy agent, Gemcitabine, is shown to phosphorylate mutp53 (R273H) at serine 15, leading to nuclear accumulation of mutp53, which increases chemoresistance [41,47]. Do et al. [46] also demonstrate that several TP53 mutants including R175H and R248W bind to Ets2, which upregulates expression of TDP2, a 5′-tyrosyl DNA phosphodiesterase involved in DNA repair, leading to etoposide resistance. 

Cancer cells are frequently addicted to mutp53, since knockdown of TP53 mutants attenuates malignant properties of cancer cells and tumor development in mice [48]. Tumor suppression induced by mutp53 knockdown may also be caused by reactivation of proteins (e.g., TP63, TP73) which are inhibited by mutp53. These provide rationale to identify mutp53 degraders/depleters for mutp53-targeted cancer therapy. Inhibitors of HSP90 have been shown to attenuate lymphoma and colon cancer progression via mutp53 (R248Q, mouse R172H) degradation in spontaneous mouse models [49,50]. Additionally, statins are shown to reduce mutp53 [29,51]. Parrales et al. [29] show that several statins including lovastatin and atorvastatin promote degradation of mainly misfolded or conformational mutp53 by inhibiting its interaction with HSP40 (DNAJA1) in a CHIP-ubiquitin ligase dependent manner, resulting in inhibited tumor malignancy and progression in xenograft mouse models. It should be noted that levels of DNA contact mutp53 also reduce, to some extent, at a higher concentration of lovastatin. Similarly, Ingallina et al. [51] report that cerivastatin induces MDM2-mediated degradation of several TP53 mutants by inhibiting interaction between mutp53 and HSP90, leading to reduce colony formation of MDA-MB-231 cells [51]. Importantly, some of HSP90 inhibitors and statins are in cancer clinical trials (https://clinicaltrials.gov/). 

Many TP53 mutants are structurally altered, especially in the DNA binding domain, thereby losing the DNA binding potential [52]. Researchers have attempted to identify compounds that restore wtp53 activity in cells expressing only mutp53 through cell-based (PRIMA-1, MIRA1, thiosemicarbazones, chetomin, RITA, NSC319726/ZMC1, p53R3, SCH529074, WR1065) or structure-based (stictic acid, PhiKan083, CP-31398, PK7088) screens [39]. APR-246 is a methylated analog of PRIMA-1, while MIRA2/3 are analogs of MIRA1. One of the potential mechanisms of reactivator compounds is the promotion of refolding of wtp53 structure directly (e.g., refolding promotion by stabilization of the TP53 core domain) or indirectly (e.g., refolding through molecular chaperones). They may also bind to and stabilize a residual population of mutp53 with pre-existing wtp53 conformation [53,54,55]. Alternatively, some reactivators may increase transcriptional activities of TP53 family members, TP63 and TP73, since both TP63 and TP73 recognize the same DNA sequence as TP53. Most of these compounds successfully upregulate some of TP53 target genes; however, only a few compounds, including PRIMA-1, p53R3, and chetomin, are shown to restore mutp53’s ability to bind to the TP53-responsive element [56,57,58]. Although the exact mechanisms of mutp53 reactivation remain to be elucidated, these reactivators have been shown to efficiently induce cell cycle arrest or apoptosis in vitro and in vivo to suppress tumor malignancy and progression [39,53]. Hence, reactivators have been shown to successfully overcome the mutp53 GOF activity by restoring wtp53 function. It should be noted that APR-246 is currently in Phase II clinical trials for cancers carrying mutp53 (https://clinicaltrials.gov/). 

Accumulated studies demonstrate efficacy of mutp53 degraders/depleters and reactivators to inhibit tumor progression in vitro and in vivo; however, it remains unclear whether these compounds cause PTMs of mutp53 and if the PTMs induced by them play crucial roles in mutp53 degradation or reactivation. 

## 3. PMTs of Mutp53

**Figure 3:**
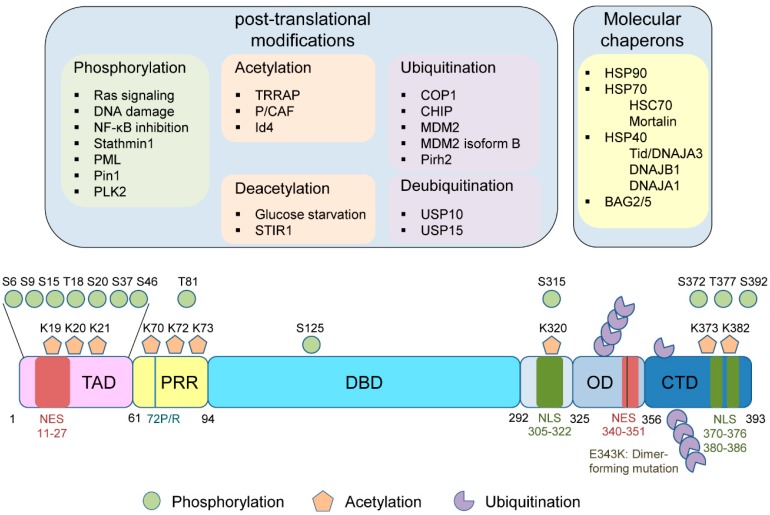
Upstream factors regulating mutant TP53 (mutp53) stability and activity, including post-translational modifications (PTMs) and molecular chaperones, as well as single nucleotide polymorphism 72 (SNP72) and dimer-forming mutations, in mutp53. TAD: Transactivation domain, PRR: Proline-rich region, DBD: DNA binding domain, NLS: Nuclear localization signal, OD: Oligomerization domain, NES: Nuclear export signal, CTD: C-terminal domain.

Wtp53 is post-translationally modified following a variety of genotoxic and cellular stresses at approximately 15% of amino acid residues, including serine, threonine, and lysine residues, through phosphorylation, acetylation, and ubiquitination [59,60,61]. Mutp53 is also modified similarly, but the biological consequences are completely different from those by wtp53. This is mainly because a stress stabilizes and activates wtp53 resulting in suppression of cancer progression as a tumor suppressor, whereas the same stress can stabilize mutp53 leading to exacerbated tumor malignancy by the oncogenic GOF. It is also possible that mutp53 is modified through other PTMs like wtp53, including methylation [62], SUMOylation [62], neddylation [62], ADP ribosylation [63], methionine oxidation [64,65], cysteine alkylation [66], and tyrosine nitration [67]; however, little is known about these effects on the stability, subcellular localization, and activity of mutp53 in cancer cells. Figure 3 summarizes PTMs, molecular chaperones, and amino acid residues which are potentially involved in mutp53 stability/activity.

### 3.1. Phosphorylation

Phosphorylation of proteins is one of the major mechanisms for regulating protein stability or function and transmitting signals in the cell. Following various stresses, wtp53 is phosphorylated at several serine/threonine residues, including serine 15 (S15), threonine 18 (T18), serine 20 (S20), serine 46 (S46), and serine 392 (S392) [59]. Intriguingly, these phosphorylation sites are rarely mutated in cancers [68]. Although mutp53 is also phosphorylated, the effects on mutp53 stability and activity are less understood as compared with those on wtp53 (Figure 3) [69,70]. 

Ullrich et al. [71] report that in glioma and adenocarcinoma cell lines, TP53 mutants (M237I and V143A) are less phosphorylated at S15 but are more phosphorylated at S392 as compared to wtp53. Minamoto et al. [72] also observe that mutp53 is phosphorylated at various sites including S9, S15, S20, S37, S46, T81, S125, S372, and S392 in human tumors. Intriguingly, the site and extent of phosphorylation are cell line-dependent, although S15, T81, and S392 appear to be more frequently phosphorylated among these serine and threonine residues. Below are studies that show functional changes in mutp53, accompanied with the corresponding phosphorylation (Table 1). 

The N-terminal serine residues of mutp53 are phosphorylated at multiple sites. Adorno et al. [73] show that activated Ras signaling in MDA-MB-231 cells promotes phosphorylation at S6 and S9 of mutp53 (R280K), promoting formation of a complex consisting of mutp53, Smad2, and TP63 to inhibit metastasis suppressor function of TP63. 

Another well-studied phosphorylated serine residue at the N-terminus of mutp53 is S15, a key serine residue to activate wtp53 following genotoxic stress [108]. Melnikova et al. [109] find constitutive S15 phosphorylation in several TP53 mutants (V154A/R155C, H176Y, R270C, E283K) through ERK1/2 activation, using multiple UV-induced mouse skin tumors and their derived cell lines. In agreement, Song et al. [74] suggest that constitutively activated DNA damage signaling in mutp53-harboring tumors could account for stabilization of mutp53 via chronic S15 phosphorylation. However, Li et al. [94] show that several mutp53-carrying cell lines, including EB2, T47D, and DU145, lack constitutive phosphorylation of S15, although genotoxic stress induces the phosphorylation. Moreover, no correlation is found between S15 phosphorylation and the levels or localization of mutp53 in three breast cancer cell lines, HCC2157 (R248Q), MDA-MB-468 (R273H), and T47D (L194F) [110]. Intriguingly, Zerbini et al. [75] observe that inhibition of NF-κB by IκB overexpression in DU145 cells results in S15 phosphorylation of mutp53 (P223L/V274F) via GADD45α-mediated JNK1 activation, leading to potential restoration of wtp53 activity including increased p21 levels, DNA binding potential, and apoptosis. Conversely, Sugikawa et al. [111] show that a mutation at S15 or S315 to alanine in R175H mutp53 partially restores wtp53 function (apoptosis induction and DNA binding potential) in Saos2 osteosarcoma cells. Thus, phosphorylation of S15 in mutp53 may alter the structure or activity of mutp53, depending on cellular contexts or experimental conditions used.

Additionally, stathmin 1 (also known as oncoprotein 18 or metablastin) which plays a role in microtubule destabilization and dynamics contributes to mutp53 stability with phosphorylation at S15 and S37 [76]. Knockdown of stathmin 1 leads to reduced mutp53 stability, decreased BUB1 expression due to attenuated GOF activity, and inhibited viable cell proliferation, in multiple mutp53-expressing ovarian cancer cell lines including TOV112D (R175H) and MDAH-2774 (R273H). This is mainly due to stathmin 1’s effects on enhancing interaction of mutp53 with DNA-PK, phosphorylation of mutp53 at S15 and S37 by DNA-PK, and mutp53 stabilization [76].

Besides S15, T18 and S46 are phosphorylated on wtp53, which is enhanced by PML [112,113,114]. PML also interacts with mutp53 (R175H, R273H, R273H/P309S) and is required for proliferation and colony formation of cancer cells bearing mutp53, suggesting that PML supports mutp53 GOF [77]. However, it remains unclear whether PML indeed promotes phosphorylation of mutp53 and which phosphorylation site induced by PML is required for enhanced proliferation and colony formation by mutp53.

S46 of wtp53 is known to be required for TP53-mediated apoptosis [115,116,117]. Peptidyl-prolyl cis/trans isomerase (PPIase, namely Pin1) isomerizes phospho-Serine/Threonine-Proline motifs, and Pin1-mediated isomerization of phosphorylated S46-P47 site in TP53 unleashes TP53’s apoptotic potential upon DNA damage, by inducing its dissociation from the apoptosis inhibitor iASPP [118]. In mice carrying homozygous *TP53^R172H^* mutations, concomitant homozygous deletion of *Pin1* attenuates tumor progression [78]. Pin1 also enhances migration and lung colonization of MDA-MB-231 cells in a manner dependent on mutp53. Moreover, Pin1 accelerates the inhibitory interaction of mutp53 with TP63, leading to inhibited metastasis suppressor function of TP63 and hence enhancing tumor aggressiveness [78]. Thus, in cells having wtp53 Pin1 supports TP53’s tumor suppressor function by promoting apoptosis, while Pin1 enhances mutp53 GOF and tumor progression in cells expressing mutp53. It remains unclear whether observed phenotypes by Pin1 are mediated through its detection of phosphorylated S46-P47 site in mutp53.

C-terminal serine residues of mutp53 are also phosphorylated. Polo-like kinase 2 (PLK2), which is activated by DNA damage, phosphorylates mutp53 (R175H, R273H) at T377, leading to enhanced binding of mutp53 with p300, increased acetylation of mutp53, and induction of the GOF activity including increase in cell proliferation, NF-Y’s transcriptional activity on cyclin A, cyclin B, cdk1, and cdc25C, and Adriamycin resistance [79].

Additionally, Matsumoto et al. [80] reveal S392 phosphorylation in over 50% of TP53-positive (indicative of TP53 mutation; 90/137 tumors) esophageal squamous cell carcinomas (ESCCs) by immunohistochemistry. Importantly, phosphorylation of S392 is correlated with high levels of Ki67 staining, lymphatic invasion, and poor prognosis for patients with stage II and III advanced tumors. In their study, only three cases (2.2%) show phosphorylation at S15. These results suggest that S392 phosphorylation in mutp53 may contribute to ESCC tumor progression [70,80]. It has also been proposed that S392 phosphorylation in mutp53 could enhance tetramer formation of mutp53, which may enhance hetero-oligomerization with wtp53 showing the dominant-negative effects (and likely GOF as well) and cell proliferation in aggressive urothelial transitional cell carcinomas (TCCs) [81]. To support this idea, Gillotin et al. [119] report that a non-phosphorylatable mutation at S392 (S392A) in R175H mutp53 reduces the protein half-life. Intriguingly, S392A mutation does not affect the half-life of R248W mutp53 [119]. Moreover, Yap et al. [120] demonstrate that S392A mutation in two hotspot TP53 mutants (R175H and R248W) transforms rat embryonic fibroblasts in cooperation with Ha-Ras oncogene more potently than R175H and R248W TP53 mutants, suggesting that the non-phosphorylated form of mutp53 at S392 has increased oncogenic activity. Thus, the role of S392 phosphorylation in mutp53 stability and its oncogenic activity may be dependent on types of TP53 mutations and cellular contexts.

Overall, phosphorylation of wtp53 stabilizes and enhances the function of wtp53 as a transcription factor and a tumor suppressor. On the other hand, phosphorylation of mutp53 results in context-dependent changes in the stability and oncogenic GOF. Further detailed studies are needed to clarify the role of each phosphorylation site in mutp53 GOF.

### 3.2. Acetylation

CBP/p300, P/CAF (p300/CBP-associated factor), and Tip60 are well-documented acetyltransferases for wtp53 which enhance wtp53 transcriptional activity [121], while HDAC1 and SIRT1 deacetylate and inhibit the wtp53 function [122,123]. However, unlike wtp53, little is known about acetylation of mutp53 and the role in the mutp53 GOF activity.

Thirteen lysine (K) residues on wtp53 are reported to be acetylated. Three (K120, K164, K292) are in the DBD, two (K305, K320) are in the domain containing a nuclear localization signal (NLS), and eight (K351, K357, K370, K372, K373, K381, K382, K386) are located in the C-terminal regulatory domain including a tetramerization/oligomerization domain, a nuclear export signal (NES), and a negative regulatory domain (Figure 3) [124,125]. Notably, mutations in K120, K164, and K305 residues are found in human cancers [62]. Like wtp53, most of the lysine residues subject to acetylation in mutp53 could also be modified by methylation, ubiquitination, SUMOylation, and neddylation; however, little is known about the roles of lysine methylation, SUMOylation, and neddylation in mutp53 GOF [62,124,125].

Minamoto et al. [72] report that mutp53 is hyperacetylated at K320, K373, and K382 in multiple cancer cells, including Hs578T breast cancer (V157F), U118MG glioblastoma (R213Q), DMS-92 small cell lung carcinoma (M237I), DLD1 colon adenocarcinoma (S241F), NCI-H596 lung adenocarcinoma (G245C), COLO320 colon adenocarcinoma (R248W), WiDr colon adenocarcinoma (R273H), HT-29 colon adenocarcinoma (R273H), ASPC-1 pancreatic adenocarcinoma (R273H), and Capan-2 pancreatic adenocarcinoma (R273H), as compared with acetylation on wtp53 in two fibroblast cell lines (GM00038, TIG). Warnock et al. [126] also show acetylation of K382 on R273H in multiple colon cancer cell lines (HT29, SW480, SW620). Recently, Jethwa et al. [82] have reported that TRRAP, a member of the phosphatidylinositol 3-kinase-related kinase (PIKK) family which is known to recruit histone acetyltransferases (HATs) to chromatin during transcription and DNA repair [127], increases the levels of multiple TP53 mutants through inhibition of the MDM2-proteasome axis in Burkitt lymphoma (BL-41: R248Q, BL-60: R248Q, CA-46: R248Q, DG-75: R283H/G245S, Namalwa: R248W, Raji: R213Q/Y234H, Ramos: I254D), diffuse large B-cell lymphoma (SUDHL-4: R273H), and colorectal cancer (Colo320: R248W) cell lines. Indeed, upon silencing of TRRAP, acetylation of mutp53 is significantly reduced. However, TRRAP knockdown induces arrest at G0/G1 phase of the cell cycle independent of TP53 status. Moreover, it remains unknown how much TRRAP contributes to mutp53 GOF activities in cancer. Nonetheless, these results support the idea that mutp53 acetylation plays a role in accumulation and oncogenic activity of mutp53 [60].

Intriguingly, Perez et al. [83] observe that exogenously expressed mutp53 (R175H, G245A, D281G) is acetylated at K320 and K373 by P/CAF upon treatment with TSA (trichostatin A), a deacetylase inhibitor, accompanied by increased apoptosis with enhanced DNA binding of mutp53 to the *p21* and *PUMA* promoters. Additionally, Knowell et al. [84] find that Id4-induced interaction between mutp53 and CBP/p300 results in increased acetylation at K320 and K373 on mutp53 (P223L/V274F), accompanied by apoptosis with upregulation of p21, BAX, and PUMA in DU145 cells. These results suggest the possibility that acetylation at K320 and K373 in mutp53 could alter the structure of mutp53 to restore wtp53 activity [128].

As mentioned in the previous section, glucose restriction triggers mutp53 degradation via autophagic cell death in several mutp53-carrying cancer cell lines, including TOV112D (R175H), MDA-MB-231 (R280K), T47D (L194F), BT-20 (K132Q), and PANC1 (R280T) [36]. Intriguingly, mutp53 (G245A) with acetylation-mimic (glutamine: Q) mutations at six lysine residues in K319, K320, K321, K370, K372, and K373 (referred to as G245A-K6Q) shows resistant to degradation and cell death induced by glucose restriction in H1299 cells, as compared with mutp53 (G245A). These results suggest that mutp53 stability is regulated by the level of glucose, and deacetylation at the C-terminal lysine residues is involved in the glucose restriction-mediated mutp53 degradation.

Moreover, Yi et al. [85] observe that deacetylation of mutp53 results in reduced levels and oncogenic activity of mutp53; YK-3-237, a small molecule compound which activates a deacetylase SIRT1, reduces acetylation of K382 of mutp53, using triple-negative breast cancer (TNBC) cell lines, BT549 (R249S), MDA-MB-468 (R273H), HS578T (V157F), and SUM149PT (M237I). This leads to reduced mutp53 level, induction of apoptotic cell death, and G2/M cell cycle arrest, with induction of wtp53 target genes (*PUMA*, *NOXA*), suggesting reactivation of wtp53 activity through deacetylation. In support of their findings, Zhang et al. [129] demonstrate that activated SIRT1 with phosphorylation at S47 serves as a prognostic factor for a longer relapse-free survival (RFS) in patients with hepatocellular carcinoma (HCC) carrying mutp53.

Thus, some lysine residues contribute to stabilization and activation of GOF mutp53, while other residues can alter the structure of mutp53 to restore wtp53 activity upon acetylation or deacetylation. Detailed analyses for each lysine residue in mutp53 are required to understand the exact role of mutp53 acetylation in its stabilization, GOF activity, and restoration of wtp53 activity.

### 3.3. Ubiquitination

Ubiquitination of mutp53 plays a crucial role in the stability and subcellular localization of mutp53, which impacts its GOF activities. Polyubiquitination of mutp53 leads to its degradation, while its monoubiquitination may alter subcellular localization of mutp53 likewise wtp53 [130,131]. There are many ubiquitin ligases and deubiquitinases that regulate ubiquitination of wtp53 to alter the stability, activity, and subcellular localization of wtp53, which is described in detail by other excellent review articles [132,133]. However, studies addressing ubiquitination of mutp53 are much less published as compared to those of wtp53.

Lukashchuk and Vousden [86] reveal that MDM2 retains the ability to interact with and induce degradation of mutp53 independently of the N-terminal interaction. However, some TP53 mutants including R175H appear to be highly ubiquitinated by other ubiquitin ligases, such as COP1 (constitutively photomorphogenic 1) and CHIP, in tissue culture [86]. Additionally, they show that R175H mutp53 is ubiquitinated, but not degraded, and localizes to the cytoplasm in *MDM2^−/−^TP53^−/−^* cells, whereas R273H mutp53 which is not ubiquitinated in the same cells is present in the nucleus [86]. Similarly, Nie et al. [87] observe that unfolded/misfolded mutp53 (detected by pAb240; C135Y, V143A, H179E) is more efficiently ubiquitinated and localizes to the cytoplasm to a greater extent than DNA contact mutp53 which retains wtp53 conformation (detected by pAb1620; R248W).

Recently, Frum et al. [88] report that phosphorylation of mutp53 (R273H) at S15 by ataxia–telangiectasia mutated (ATM) kinase following DNA damage results in monoubiquitination by MDM2, but not polyubiquitination, while ATM inhibition restores MDM2’s ability to polyubiquitinate and degrade mutp53. These results suggest that G2M checkpoint activation of the cell cycle accumulates mutp53 by promoting monoubiquitination by MDM2, rather than polyubiquitination [88].

MDM2 has more than 40 splice variants, but it is unclear whether all variants are translated and have functional effects on TP53 [134]. Several spliced isoforms, such as isoform A, B, and C, are detected and co-expressed with full-length MDM2 (MDM2-FL) in tumors. MDM2 isoform B (MDM2-B) is one of the most studied isoforms lacking the TP53-binding domain but retaining the C-terminal domain to interact with MDM2-FL [135,136,137]. Zheng et al. [89] demonstrate that MDM2-B promotes mutp53 accumulation in multiple human cancer cell lines endogenously or exogenously expressing R175H, R248W, R273H, Y220C, and S241F. This accumulation is caused by MDM2-B’s ability to bind with and inhibit MDM2-FL which mediates mutp53 degradation. Using *TP53^R248W/−^* HCT116 cells, they also show that MDM2-B enhances mutp53 GOF including tumor growth and metastasis in vivo. Additionally, MDM2-B is overexpressed in *TP53^R172H/R172H^* mouse lymphomas. Thus, it is postulated that any proteins which inhibit MDM2 activity could stabilize mutp53 to increase the GOF activities.

CHIP is a cofactor that interacts with HSC70 and accelerates ubiquitin-dependent degradation of chaperone substrates through its carboxyl-terminal U-box [138]. Mutp53 (R175H) degradation by CHIP is shown by Esser et al. [139]. HSP90 inhibition also induces CHIP-mediated degradation of mutp53 [94]. Our group has recently demonstrated that cholesterol-lowering drugs, “statins,” inhibit binding between conformational/misfolded mutp53 (R156P, R175H, Y220C) and DNAJA1, a member of HSP40, leading to CHIP-mediated nuclear export and degradation of mutp53 [29]. Intriguingly, knockdown of DNAJA1 or mevalonate kinase also induces CHIP-mediated ubiquitination and degradation of mainly conformational/misfolded mutp53, similarly to statin treatment [29]. Moreover, Maan et al. [30] report that CHIP induces ubiquitination and degradation of aggregating TP53 mutants (R110P, R175H), but not non-aggregating mutants (R248W, R273H), both in normoxia and hypoxia. The observed ubiquitination and degradation of aggregating TP53 mutants by CHIP are mediated via K63-linked polyubiquitination and are dependent on autophagy [30]. These results may suggest that CHIP mainly targets unfolded/misfolded aggregating TP53 mutants for degradation.

Another ubiquitin ligase which can ubiquitinate and degrade wtp53 is Pirh2. Intriguingly, arsenic trioxide (ATO) induces Pirh2-mediated degradation of multiple TP53 mutants in SW480 (R273H), MiaPaCa-2 (R248W), HaCaT (H179Y/R282W) cells, as well as HCT116 cells expressing R175H and R273H [90].

In summary, mutp53 is ubiquitinated by MDM2 and other ubiquitin ligases. Unfolded/misfolded mutp53 may be ubiquitinated by multiple ubiquitin ligases such as MDM2 and CHIP, while ubiquitination of DNA contact mutp53 is mainly executed by MDM2. Additionally, ubiquitinated mutp53 appears be exported to the cytoplasm for degradation likewise wtp53. Thus, ubiquitination and degradation of mutp53 may be dependent on its binding to molecular chaperones and their associated ubiquitin ligases.

Deubiquitinases (DUBs) are a group of proteases that cleave ubiquitin from proteins. USP10 is a cytoplasmic ubiquitin-specific protease and is shown to deubiquitinate wtp53 to reverse MDM2-induced wtp53 ubiquitination, nuclear export, and degradation [91]. Knockdown of USP10 results in reduction in wtp53 levels and wtp53-mediated apoptosis. This group also addresses the question of whether USP10 acts on mutp53. USP10 levels are positively correlated with mutp53 in renal cell carcinoma (RCC) tissues; USP10 is overexpressed in RCC having mutp53, whereas USP10 levels are undetectable in RCC tissues with wtp53. In the 786-O RCC cell line, USP10 overexpression inhibits MDM2-mediated mutp53 ubiquitination leading to stabilization of mutp53, while USP10 downregulation increases mutp53 ubiquitination to decrease its stability. Furthermore, overexpression of USP10 in 786-O cells increases levels of mutp53, leading to enhanced colony formation and proliferation, which is nullified by depletion of mutp53. Thus, increased USP10 levels would be beneficial to progression of cancer carrying mutp53, whereas in cancer cells with wtp53 USP10 acts as a tumor suppressor [91]. However, it is unclear whether USP10 antagonizes ubiquitination of mutp53 by other ubiquitin ligases, USP10 alters subcellular localization of mutp53, and USP10 plays a role in deubiquitination of different TP53 mutants.

USP15 is another deubiquitinase that is reported to increase the stability of MDM2 and hence reduce wtp53 activity to inhibit apoptosis in cancer cells [140]. However, Padmanabhan et al. [92] have recently shown that MCB-613, a small molecule stimulator of steroid receptor coactivators (SRCs), enhances nuclear export, ubiquitination, and lysosome-mediated degradation of R175H mutp53, but not R273H, through depletion of USP15 protein via a post-translational mechanism independent of MDM2, leading to reduced viability of ovarian cancer cells carrying R175H (TYK-Nu, TOV112D). A DUB inhibitor (NSC632839) and USP15 knockdown phenocopies biological effects of MCB-613. However, it remains unclear why only R175H, but not R273H, is degraded by USP15 depletion and how exactly MCB-613 reduces USP15 levels independent of SRC stimulation.

Thus, several ubiquitin ligases and deubiquitinases can regulate ubiquitination and degradation of both wtp53 and mutp53; however, it remains unclear whether a ubiquitin ligase which ubiquitinates both wtp53 and mutp53 modifies the same lysine residues, if different ubiquitin ligases modify the same lysine residues, and which lysine residues are involved in subcellular localization of mutp53. It is also unknown if other ubiquitin-like modifications such as neddylation and SUMOylation occur in mutp53 and whether these modifications alter the stability, GOF activities, and subcellular localization of mutp53.

## 4. Molecular Chaperones

Endogenous and environmental stresses, such as heat shock, oxidative stress, inflammation, infection, chemicals, and irradiation, significantly impact behavior of the cell. Heat shock proteins (HSPs) play central roles in responding to these stresses as molecular chaperones. Molecular chaperones interact with a variety of proteins to promote their proper folding in order to prevent generation of unfolded/misfolded or damaged proteins, causing them to be refolded or degraded [141].

HSP family proteins, including HSP90, HSP70 (DnaK homologue), and HSP40 (DnaJ homologue), play major roles in protein folding, protein maturation, protein localization, proteolysis, and cell signaling [141]. There are many studies documenting physical and functional interactions of HSP90 and HSP70 with wtp53 [93,142,143,144]. HSPs are also known to bind with mutp53 to refold, stabilize, or degrade it [93,144,145,146,147,148].

HSP90 plays a crucial role in stabilizing mutp53. Geldanamycin, a HSP90 inhibitor, alters the physical association of mutp53 with the HSP90 complex, leading to reduced protein half-life of several TP53 mutants via ubiquitination and proteasomal degradation, in multiple cancer cell lines, including T47D (L194F), MDA-MB-468 (R273H), and SKBR3 (R175H) [149,150,151]. HSP90 is shown to form a complex with MDM2 and mutp53 (L194F, S241F, R273C, R273H) to block ubiquitination of both MDM2 and mutp53, while geldanamycin induces MDM2-mediated mutp53 degradation [93]. Moreover, Li et al. [94] demonstrate that HSP90 forms a complex with mutp53 to prevent mutp53’s aggregation by inhibiting the E3 ligase activity of MDM2 and CHIP in multiple cancer cells, including MDA-MB-468 (R273H), MDA-MB-231 (R280K), DU145 (P223L/V274F), T47D (L194F), SW480 (R273H/P309S), SKBR3 (R175H), and 5637 (R280T). Additionally, Li et al. [95] report that SAHA (suberoylanilide hydroxamic acid), a HDACi, causes cell death preferentially in cancer cells carrying mutp53 (SW480, DU145, T47D, MDA-MB-231, ES2, and SKBR3) and sensitizes MDA-MB-231 and T47D cells to a chemotherapy agent, Camptothesin. This is due to degradation of mutp53 by SAHA, which is likely caused by hyperacetylation and inhibition of HSP90 through inactivation of HDAC6 by SAHA [152]. Thus, SAHA ultimately inhibits HSP90 activity, releasing mutp53 from the HDAC6-HSP90-mutp53 complex, enabling mutp53 degradation by MDM2 and CHIP [95]. Importantly, in vivo evidence showing that cancer cells are addicted to mutp53 is provided by Alexandrova et al. [50], using HSP90 inhibitors; pharmacological inhibition of HSP90 by ganetespib results in degradation of mutp53 in lymphomas and prolongs survival of *TP53^R172H/R172H^* and *TP53^R248Q/−^* mice with minimal effects on *TP53-null* mice. Recently, Ingallina et al. [51] observed that stiffness in the extracellular matrix stimulates RhoA-dependent remodeling of filamentous actin and actomyosin contractility which can accumulate mutp53 (V157F, M273I, R280K, mouse R172H) through the HDAC6-HSP90 axis. Thus, inhibition of RhoA activity through inhibition of the mevalonate–RhoA axis by an HMG-CoA-R inhibitor (cerivastatin) or an inhibitor of geranylgeranyltransferase type I (GGTI-298) reduces HDAC6 activity and hence induces hyperacetylation of HSP90, leading to dissociation of mutp53 from HSP90 and degradation of mutp53 by MDM2 (R175H, L194F, M237I, R249S, R273H).

HSP70 is another chaperone which is involved in mutp53 stabilization or degradation. Muller et al. [96] show that HSP70/HSC70 (heat-shock cognate protein of 70 kDa) selectively recognizes unfolded/misfolded TP53 proteins and promotes its CHIP-dependent ubiquitination and degradation when HSP90 activity is inhibited and hence mutp53 folding is blocked (R175H, R273H). On the other hand, Wiech et al. [97] observe that HSP70, whose levels are commonly high in cancers, accelerates CHIP-mediated degradation of mutp53 (R175H), whereas HSP70 partially inhibits MDM2-mediated ubiquitination and degradation of exogenous mutp53 (V143A, R175H) to enhance formation of nuclear aggregates. Intriguingly, HSC70 inhibits the process of nuclear aggregation of mutp53, suggesting that the mechanism of HSP70 activity towards mutp53 could be different from that of HSC70. Moreover, Vakifahmetoglu-Norberg et al. [35] reveal that mutp53, but not wtp53, binds to a cytosolic HSC70 when ES2 (S241F) cells are treated with spautin-1, a small molecule inhibitor of autophagy, under confluency or nutrient deprivation conditions. This experimental condition induces chaperone-mediated autophagy (CMA) which facilitates nuclear export of mutp53 and promotes interaction of mutp53 with HSC70, leading to mutp53 degradation through the lysosome. However, Finlay et al. find that conformational mutp53 preferentially binds to HSC70, but this HSC70-mutp53 complex increases the protein half-life of mutp53 [153]. Such inconsistent roles of HSP70 and HSC70 in mutp53 stability may be dependent on cellular context or experimental settings.

Mortalin is a member of mitochondrial HSP70 and is also known as mtHSP70/Grp75 (mot-2). Mortalin is involved in mitochondrial biogenesis regulating mitochondrial import of nuclear-encoded proteins [154]. Mortalin is also shown to sequester wtp53 to the cytoplasm [155]. Recently, Lu et al. [98] reported that mortalin binds to mutp53, and knockdown of mortalin results in nuclear translocation of mutp53 and apoptosis in an HCC cell line, PLC/PRF/5. This is not observed in MIHA and HepG2 (wtp53) cell lines, and apoptosis induced by mortalin knockdown is nullified by a TP53 inhibitor (PFT-µ) or knockdown of mutp53. These results suggest that mortalin-knockdown-mediated apoptosis is dependent on mutp53 [98]. However, detailed mechanisms including whether mortalin knockdown restores structure and activity of wtp53 remain unclear.

HSP40 is another chaperone that binds with conformational or misfolded mutp53 (R175H) [156]. Mass spectrometry analyses also support binding of multiple HSP40 members with mutp53 [146,156]. Tid1/DNAJA3 is the tumor suppressor and forms a complex with wtp53 under hypoxia induced by an iron chelator desferroxamine (DFX), which causes wtp53 translocation to the mitochondria and apoptosis [99,157]. In multiple glioma and breast cancer cells carrying mutp53, including U373 (R273H), T47D (L194F), SKBR3 (R175H), and BT474 (E285K), overexpression of Tid/DNAJA3 restores mitochondrial localization and pro-apoptotic activities of TP53 when these cells are treated with DFX [99]. Additionally, Hiraki et al. [57] identify HSP40, specifically DNAJB1, as a factor that contributes to restoring TP53 activity of mutp53 (R175H) when cells are treated with chetomin (CTM), through a cell-based, high-throughput small molecule screen. CTM binds to HSP40 and increases the interaction between HSP40 and R175H mutp53, leading to restoration of wtp53 activity. Indeed, CTM specifically inhibits proliferation and tumor growth of multiple cancer cells expressing R175H mutp53 with upregulation of TP53 target genes including *p21*, *PUMA*, and *MDM2*, as well as mutp53’s binding to the TP53-resposible elements of these genes. Moreover, CTM restores MDM2’s ability to induce mutp53 degradation. Additionally, Tracz-Gaszewska et al. [100] observe that DNAJB1 and HSP70 facilitate binding of mutp53 (V143A, R158L, R175H, Y220C, G245S, D281G, R282W) to TAp73α. Another HSP40 member that plays a role in stabilization of mutp53 is DNAJA1. Our group reveals that DNAJA1 binds to and stabilizes mainly conformational or misfolded mutp53 (R156P, R175H, Y220C) by competitively binding to CHIP ubiquitin ligase [29]. Intriguingly, statins inhibit the mutp53-DNAJA1 binding to induce degradation of conformational/misfolded mutp53 by CHIP [29].

BCL2-associated athanogene (BAG) family proteins are co-chaperones that interact with mutp53. Through mass spectrometry analyses, two BAG family proteins, BAG2 and BAG5, are found to bind with mutp53 (mouse R172H) and promote its GOF activity [101,102]. Yue et al. [101] show that BAG2 binds to mouse R172H mutp53 in mouse embryonic fibroblasts (MEFs) and multiple human cell lines including *TP53^R248W/−^* HCT116 cells as well as H1299 and Saos2 cells exogenously expressing R175H, R248W, and R273H mutp53. They observe that BAG2 inhibits MDM2’s activity on mutp53 and accumulate the protein levels of mutp53. Furthermore, BAG2 knockdown enhances sensitivity of mutp53-expressing cells to 5-Fluorouracil, a chemotherapy drug, and reduces migration, tumor growth, and lung colonization of *TP53^R248W/−^* HCT116 cells. Moreover, BAG2 is elevated in a diverse range of human cancers, including colorectal cancers, lung cancers, breast cancers and sarcomas, while high levels of BAG2 are associated with increased mutp53 levels and poor prognosis in these cancer patients [101]. Later, the same group also found that another BAG member, BAG5, interacts with several mutp53 (R175H, R248W, R273H, mouse R172H) to inhibit ubiquitination and degradation of mutp53 by MDM2 and CHIP E3 ubiquitin ligases [102]. This interaction results in mutp53 accumulation and enhanced GOF activities including cell proliferation, migration, chemoresistance, and tumor growth. Interestingly, BAG5 cooperates with BAG2 on cell migration and chemoresistance. Similar to BAG2, BAG5 is overexpressed in many human tumors (colorectal cancers, lung cancers, breast cancers and skin) with a positive correlation with poor prognosis of breast cancer patients [102].

Thus, multiple chaperone and co-chaperone proteins play crucial roles in mutp53 stability and GOF activities as well as restoration of wtp53 structure and activity. Currently, only HSP90 inhibitors are in clinical trials for cancer therapy. Development of inhibitors for other HSPs and chaperones/co-chaperones would greatly help understand mechanisms behind restoration of wtp53 activity and mutp53 stabilization/GOF and may also advance current therapeutic strategies for cancers carrying mutp53.

## 5. SNP at Codon 72 in Mutp53

A SNP in wtp53 at codon 72 alters localization and function of wtp53, where arginine 72 (72R)-wtp53 has greater ability to localize to the mitochondria and induce apoptosis more efficiently than proline 72 (72P)-wtp53 [158]. Evidence indicates that SNP72 in mutp53 also affects the GOF activity. Marin et al. [103] show that several TP53 mutants (V143A, V173L, R175H) carrying 72R polymorphism (72R-mutp53) more efficiently bind to TP73 to inactivate TP73-induced apoptosis than those carrying 72P (72P-mutp53) using exogenously overexpressed TP53 mutants in Saos2 cells. Similarly, Bergamaschi et al. [104] find that many 72R-mutp53, including V173L, R175H, C176Y, H179R, Y220C, C242Y, G245S/D, R249S, R282W, and R273C, show higher efficiency on inhibition of apoptosis induced by chemotherapy drugs (cisplatin, Taxol, doxorubicin), as compared to 72P-mutp53. This is mainly caused by inhibitory binding of mutp53 with TP73. Interestingly, SNP72 in some mutp53, including P142L, P152Q, R158G, and A161V, has minimal impact on cisplatin-induced apoptosis [104]. Although several studies observe that mutp53 (R175, Y220C, R248W, D281G) efficiently interacts with TP73, direct comparison of binding affinity to TP73 is not made between 72P- and 72R-mutp53 [159,160,161,162]. Vikhanskaya et al. [105] also show that 72R polymorphism in several TP53 mutants (R175H, G245S, R248W, R249S, R273H, R282W) does not show any significant differences in resistance to many chemotherapy drugs, including cisplatin, etoposide, gemcitabine, vinblastine, and Taxol, when compared with 72P-mutp53. Only the cells expressing 72R-R249S show more resistance to doxorubicin than those with 72P-R249S. Differences among these studies could be due to differences of cell types or experimental conditions used.

Recently, Basu et al. [106] reported that 72R-mutp53 (R175H, R273H) shows higher invasive and metastatic potential than those with 72P-mutp53, by overexpressing 72P- or 72R-mutp53 in *TP53-null* H1299 or PC3 cells, as well as by comparing results between two colon cancer cell lines, HT29 (72P-R273H) and SW620 (72R-R273H). They reveal that 72P-mutp53 more efficiently binds to and inhibits activity of PCG-1α, a master regulator of mitochondrial function, as compared with 72R-mutp53. As a result, cancer cells carrying 72R-mutp53 have increased mitochondrial function, leading to enhanced metastatic capacity. Moreover, 72R-mutp53 is correlated with poorer outcomes in human breast cancer, as compared with 72P-mutp53 [106]. Further studies, specifically in vivo studies using genetically engineered Hupki (human TP53 knockin) mouse models in combination with TP53 mutations, are required to obtain the physiological role of SNP72 of each TP53 mutant in mutp53 GOF and tumor progression [163,164].

## 6. Dimer-Forming Mutp53

Oligomerization of TP53 has been implicated in TP53’s DNA binding/transcriptional activity, ubiquitination/degradation, and interactions with TP53 binding partners [165]. Recently, Prives’s group has shown that the N-terminal domain of MDM2 binds to the C-terminal domain of dimer-forming TP53 in which a mutation is inserted in the TP53 oligomerization domain (E343A/K, L344A, L347T, E348A), leading to TP53 degradation [107]. Additionally, the dimer-forming TP53 is prone to MDM2-mediated nuclear export. The dimer-forming TP53 is partially unfolded and is degraded through ubiquitin-independent degradation by the 20S proteasome, but not 26S proteasome. Moreover, E343K mutation in two hotspot R175H and R248W mutp53 results in enhanced degradation of mutp53 by MDM2. Biologically, E343K mutation in R175H mutp53 leads to reduced R175H-mediated migration of H1299 cells [107]. This study may provide novel opportunities of targeting mutp53 to cancer therapy. Further studies required include clarification of in vivo physiological roles of dimer-forming TP53 in tumor progression using animal models, elucidation of the roles of naturally occurring TP53 mutations in the oligomerization domain in dimer formation, and identification of signals which impact oligomerization of mutp53.

## 7. Conclusions

This article has summarized multiple factors that impact levels, subcellular localization, and GOF activities of mutp53, as well as those inducing reactivation of wtp53 or depletion of mutp53. These include the upstream regulators of mutp53, such as genotoxic stress, PTMs, ubiquitin ligases, and molecular chaperones, as well as different sequences in mutp53 including codon 72 polymorphism and mutations in the oligomerization domain. Although studies examining upstream regulators of mutp53 GOF activities are limited, unlike wtp53, many upstream regulators of wtp53 also appear to impact mutp53 stability and GOF. However, the biological consequences are distinct between wtp53 and mutp53, which can be dependent on types of TP53 mutations, cellular context, and experimental conditions. To clarify these differences, detailed studies using various TP53 mutants in a well-controlled experimental setting should be performed in the future.

In conclusion, stability and activity of mutp53 are altered by multiple factors, some of which regulate wtp53 activity. However, the biological consequences are distinct. More studies are required to obtain clear-cut conclusion of the roles of mutp53 PTMs and SNPs in the GOF activity and cancer progression.

## Figures and Tables

**Table 1:** Regulators that alter mutant TP53 stability and activity.

Upstream Regulators	Mutant TP53	Modified Amino Acids	Samples/Cell Lines	Outcomes/Effects	References
**Phosphorylation**					
Ras signaling	R280K	S6, S9	MDA-MB-231	Mutp53/Smad2/TP63 Complex inhibit TP63’s metastasis suppressor function.	[73]
DNA damage	R248W, R273H	S15	MEFs, PANC1	Constitutively activated DNA damage could account for mutp53 stabilization or nuclear accumulation via S15 phosphorylation.	[41,47,74]
NF-κB inhibition by IκB overexpression	P223L/V274V	S15	DU145	NF-κB inhibition in DU145 cells leads to S15 phosphorylation of mutp53 via GADD45α-mediated JNK1 activation and potential restoration of wtp53.	[75]
Stathmin1	R175H, R273H	S15, S37	TOV112D, MDAH-2774	Stathmin1 enhances interaction of mutp53 with DNA-PK, phosphorylation of mutp53 at S15 and S37 by DNA-PK, and mutp53 stabilization, leading to increased viable cell proliferation.	[76]
PML	R175H, R273H, R273H/P309S	Not specified (maybe via phosphorylation at T18 and S46 in mutp53 likewise wtp53)	SKBR3, HT29, SW48	PML interacts with mutp53 and is required for proliferation and colony formation of cancer cells bearing mutp53; however, it is unclear whether PML promotes phosphorylation of mutp53 like wtp53.	[77]
Pin1	Mouse R172H, R280K	Not specified (maybe via isomerization of phosphorylated S46-P47 site in mutp53 likewise wtp53)	MEFs, MDA-MB-231	Homozygous deletion of *Pin1* attenuates tumor progression in *TP53^R172H/R172H^* mice, while Pin1 enhances migration and lung colonization of MDA-MB-231 cells in a manner dependent on mutp53 by enhancing inhibitory interaction of mutp53 with TP63; however, it is unclear whether observed phenotypes by Pin1 are mediated through its detection of phosphorylated S46-P47 site in mutp53.	[78]
PLK2	R175H, R273H	T377	H1299, SKBR3	PLK2 phosphorylates mutp53 at T377 and enhances binding of mutp53 with p300, acetylation of mutp53, and mutp53 GOF activity, including increased cell proliferation, NF-Y’s transcriptional activity, and Adriamycin resistance.	[79]
Not specified/oncogenic signaling	Not Specified	S392	Cancer tissues (esophageal squamous cell carcinoma, urothelial transitional cell carcinoma)	S392 phosphorylation in mutp53 is correlated with high levels of Ki67 staining, lymphatic invasion, and poor prognosis, as well as enhanced hetero-oligomerization with wtp53 and dominant-negative activities.	[70,80,81]
**Acetylation**					
TRRAP	R248Q, R248W, R283H/G245SS, R213Q/Y234H, I254D, R273H	Not specified	BL-41, BL-60, CA-46, DG-75, Namalwa, Raji, Ramos, SUDHL-4, Colo320	TRAPP recruits HATs to chromatin and increases acetylation and accumulation of mutp53 through inhibition of MDM2-mediated degradation.	[82]
P/CAF	R175H, G245A, D281G	K320, K373	H1299	Treatment of cells with TSA acetylates mutp53 at K320 and K373 by P/CAF to increase apoptosis with enhanced DNA binding of mutp53 to the *p21* and *PUMA* promoters.	[83]
Id4	P223L/V274F	K320, K373	DU145	Id4 increases interaction of mutp53 with CBP/p300, acetylation at K320 and K373 on mutp53, and apoptosis with upregulation of p21, BAX, and PUMA.	[84]
**Deacetylation**					
Glucose restriction	G245A	Not specified (6Q mutations in K319, K320, K321, K370, K372, K373)	H1299	G245A-K6Q shows resistance to mutp53 degradation and cell death induced by glucose restriction.	[36]
SIRT1	R249S, R273H, V157F, M237I	K382	BT549, MDA-MB-468, HS578T, SUM149PT	Activation of SIRT1 deacetylase by YK-3-237 decreases mutp53 levels with reduced acetylation at K382, leading to induction of apoptotic cell death and G2/M cell cycle arrest with induction of wtp53 target genes.	[85]
**Ubiquitination**					
COP1, CHIP (independent of MDM2)	R175H	Not specified	U2OS, H1299, *MDM2^−/−^TP53^−/−^* MEFs	Downregulation of COP1 or CHIP reduces ubiquitination of mutp53 independent of MDM2.	[86]
Not specified/MDM2-independent ubiquitination	C135Y, V143A, H179E	Not specified	U2OS, *MDM2^−/−^TP53^−/−^* MEFs	Misfolded mutp53 is more efficiently ubiquitinated and localizes to the cytoplasm to a greater extent than a DNA contact mutp53 (R248W).	[87]
MDM2	R273H	Not specified	H1048, H1299, WI38	S15 phosphorylation of mutp53 by ATM following DNA damage inhibits MDM2-mediated polyubiquitination and degradation of mutp53 with allowing its monoubiquitination and accumulation.	[88]
MDM2 isoform B (MDM2-B)	R175H, R248W, R273H, Y220C S241F	Not specified	H1299, HCT116, T47D, Huh7, DLD-1	MDM2-B binds to and inhibits full-length MDM2 (MDM2-FL)-mediated mutp53 degradation, leading to enhanced mutp53 GOF activities to promote tumor growth and metastasis.	[89]
CHIP	R156P, R175H, Y220C	Not specified	KHOS/NP, SKBR3, CAL33, BxPC3	Cholesterol-lowering drugs “statins,” knockdown of mevalonate kinase, and DNAJA1 knockdown induce CHIP-mediated nuclear export and degradation of mainly conformational/misfolded mutp53.	[29]
CHIP	R110P, R175H	Not specified	HCT116, CAL33	Aggregating TP53 mutants (R110P, R175H), but not non-aggregating mutants (R248W, R273H), are ubiquitinated and degraded by CHIP via K63-linked polyubiquitination in a manner dependent on autophagy.	[30]
Pirh2	R175H, R248W, H179Y/R282W, R273H	Not specified	SW480, MiaPaCa-2, HaCaT, HCT116	Arsenic trioxide (ATO) induces Pirh2-mediated degradation of multiple TP53 mutants.	[90]
**Deubiquitination**					
USP10	Not specified	Not specified	786-O	USP10 overexpression inhibits MDM2-mediated mutp53 ubiquitination leading to stabilization of mutp53 as well as increased colony formation and cell proliferation.	[91]
USP15	R175H	Not specified	TYK-Nu, TOV112D, SKOV3	MCB-613, a stimulator of steroid receptor coactivators (SRCs), enhances nuclear export, ubiquitination, and lysosome-mediated degradation of mutp53 (R175H), but not R273H, through inhibition of USP15, leading to reduced viability of ovarian cancer cells.	[92]
**Molecular Chaperons**					
HSP90	L194F, S241F, R273C, R273H	N/A	T47D, DLD1, C33A, MDA-MB-468	HSP90 forms a complex with MDM2 and mutp53 to block ubiquitination of both MDM2 and mutp53, while HSP90 inhibition by geldanamycin induces MDM2-mediated mutp53 degradation.	[93]
HSP90	R175H, L194F, P223L/V274F, R273H, R273H/P309S, R280K, R280T	N/A	SKBR3, T47D, DU145, MDA-MB-468, MDA-MB-231, SW480, 5637	HSP90 forms a complex with mutp53 to prevent mutp53’s aggregation by inhibiting MDM2 and CHIP activities.	[94]
HSP90 (HDAC6-HSP90-mutp53 complex)	R175H, L194F, P223L/V274F, S241F, R273H/P309S, R280K	N/A	SKRB3, T47D, DU145, ES2, SW480, MDA-MB-231	Inhibition of HSP90 activity by SAHA releases mutp53 from the HDAC6-HSP90-mutp53 complex, leading to mutp53 degradation by MDM2 and CHIP to enhance cell death by a chemotherapy agent, Camptothesin.	[95]
HSP90	mouse R172H and R248Q	N/A	T-lymphomas	Inhibition of HSP90 by ganetespib results in degradation of mutp53 in lymphomas and prolongs survival of *TP53^R172H/R172H^* and *TP53^R248Q/−^* mice with minimal effects on *TP53-null* mice.	[50]
HSP90 (HDAC6-HSP90-mutp53 complex)	R175H, L194F, M237I, R249S, R273H	N/A	SKBR3, T47D, SUM149, Mahlavu, BT549, MDA-MB-468	Inhibition of the mevalonate–RhoA axis by cerivastatin or GGTI-298 reduces HDAC6 activity, leading to HSP90 hyperacetylation and dissociation of HSP90 from mutp53, which induces degradation of mutp53 by MDM2.	[51]
HSP70/HSC70	R175H, R273H	N/A	MDA-MB-468, H1299	HSP70 selectively recognizes unfolded/misfolded TP53 proteins and promotes its CHIP-dependent ubiquitination and degradation when HSP90 is inhibited and mutp53 folding is blocked.	[96]
HSP70 (not HSC70)	V143A, R175H	N/A	*MDM2^−/−^TP53^−/−^* MEFs, H1299, SKBR3	HSP70 accelerates CHIP-mediated degradation of mutp53, whereas it partially inhibits MDM2-mediated ubiquitination and degradation of mutp53 to enhance nuclear aggregates which can be inhibited by HSC70.	[97]
HSC70	R248Q, S241F, R158Inf, R280L, G266Q, S227K, S227R, E258K, A161T, R273L, R273H, R280L, R175H, R175D, R175C, R248W, R248L, R282W, P151H, P98S, G245C, L194F	N/A	OVCAR-3, ES2, SUM159, MDA-MB-231, MDA-MB-435, HCT116	Chaperone-mediated autophagy (CMA), which is induced in cells treated with an autophagy inhibitor (spautin-1) under confluency or nutrient deprivation conditions, facilitates nuclear export of mutp53 and promotes interaction of mutp53 with HSC70, leading to mutp53 degradation through the lysosome.	[35]
Mortalin (mtHSP70/Grp75)	R249S	N/A	PLC/PRF/5	Mortalin knockdown induces nuclear translocation and apoptosis in a mutp53-dependent manner.	[98]
Tid/DNAJA3 (HSP40)	R175H, L194F, R273H, E285K	N/A	SKBR3, T47D, U373, BT474	Overexpression of Tid/DNAJA3 restores mitochondrial localization and pro-apoptotic activities of TP53 in cells treated with desferroxamine (DFX).	[99]
DNAJB1 (HSP40)	R175H	N/A	H1299, CAL-33, HuCCT1, FAMPAC, KLE, TOV112D	Chetomin (CTM) binds to HSP40 and increases the interaction between HSP40 and R175H mutp53, leading to restoration of wtp53 activity and inhibition of proliferation and tumor growth of multiple cancer cells expressing R175H with upregulation of TP53 target genes.	[57]
DNAJB1 (HSP40), HSP70	V143A, R158L, R175H, Y220C, G245S, D281G, R282W	N/A	H1299	DNAJB1 and HSP70 facilitate binding of mutp53 to TAp73α.	[100]
DNAJA1 (HSP40)	R156P, R175H, Y220C	N/A	KHOS/NP, SKBR3, CAL33, BxPC3	DNAJA1 binds to and stabilizes mainly conformational or misfolded mutp53 by competitively binding to CHIP.	[29]
BAG2	mouse R172H, R175H, R248W, R273H	N/A	R172H MEFs, H1299, Saos2, HCT116	BAG2 binds to mutp53 (mouse R172H) and inhibits MDM2’s activity to accumulate mutp53, while BAG2 knockdown increases chemosensitivity and reduces tumor growth and metastasis.	[101]
BAG5	mouse R172H, R175H, R248W, R273H	N/A	R172H MEFs, H1299, Saos2, HCT116	BAG5 binds to mutp53 (mouse R172H) and inhibits ubiquitination and degradation of mutp53 by MDM2 and CHIP to accumulate mutp53 and enhance mutp53 GOF, including cell proliferation, migration, chemoresistance, and tumor growth.	[102]
**SNP**					
72P/R polymorphism	V143A, V173L, R175H	72P, 72R	Saos2	72R-mutp53 binds more efficiently to TP73 to inactivate TP73-induced apoptosis than 72P-mutp53.	[103]
72P/R polymorphism	V173L, R175H, C176Y, H179R, Y220C, C242Y, G245S/D, R249S, R282W, R273C	72P, 72R	Saos2	72R-mutp53 shows higher efficiency on inhibition of apoptosis induced by chemotherapy drugs, as compared to 72P-mutp53, mainly due to mutp53’s inhibitory binding with TP73.	[104]
72P/R polymorphism	R249S	72P, 72R	H1299	Polymorphism at codon 72 in several TP53 mutants (R175H, G245S, R248W, R249S, R273H, R282W) do not show any significant differences in resistance to many chemotherapy drugs, whereas only 72R in R249S mutp53 makes cells more resistance to doxorubicin than 72P in R249S.	[105]
72P/R polymorphism	R175H, R273H	72P, 72R	H1299, PC13, HT29 (72P-R273H), SW620 (72R-R273H)	72R-mutp53 less efficiently binds to and inhibits activity of PCG-1α, leading to higher mitochondrial function and metastatic potential than 72P-mutp53.	[106]
**Dimer**					
Mutations in the TP53 oligomerization domain	R175H, R248W	E343K	U2OS, H1299	E343K mutation in mutp53 enhances degradation of mutp53 by MDM2, leading to inhibited migration.	[107]
